# Supplementary material for: DNA Methylation of α-Synuclein Intron 1 Is Significantly Decreased in the Frontal Cortex of Parkinson’s Individuals with GBA1 Mutations
Source: Int J Mol Sci. 2023 Jan 31;24(3):2687. doi: 10.3390/ijms24032687 (PMC9917152; doi:10.3390/ijms24032687)
Supplement: Supplementary file 1 [file ijms-24-02687-s001.zip › ijms-1980849-supplementary.pdf]

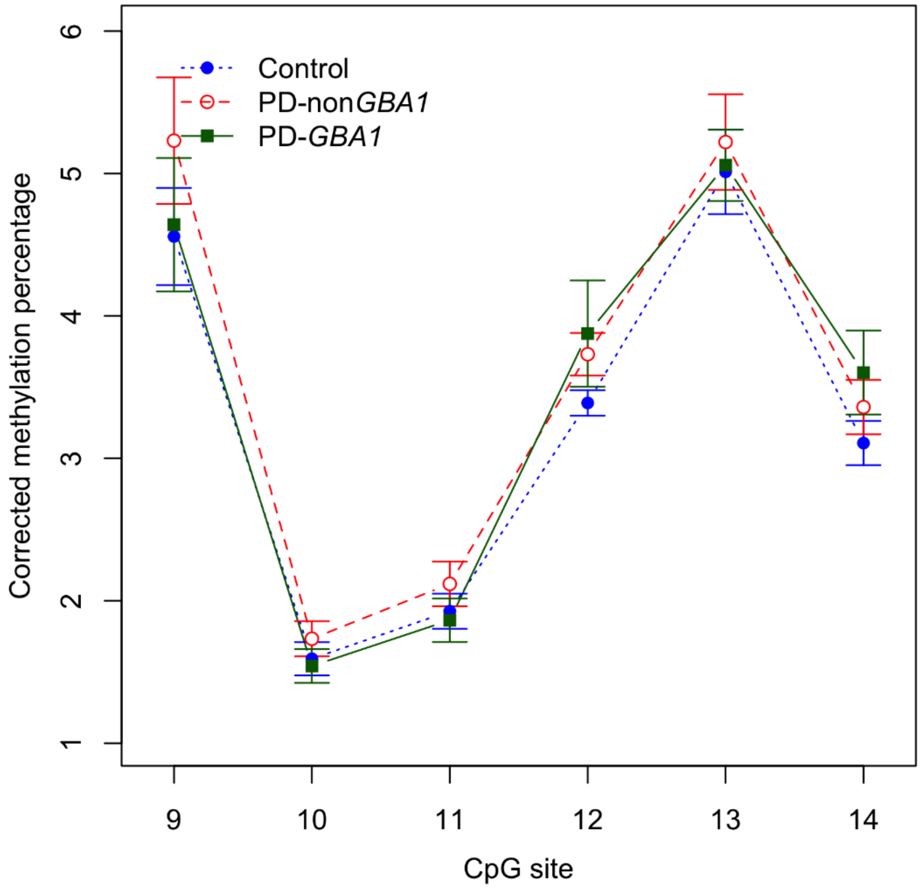


**Supplementary Figure S1: No significant DNA hypomethylation at a further six consecutive CpG sites (CpG9-14) of *SNCA* intron 1 in the frontal cortex in idiopathic PD.** The methylation profile within CpG9-14 of *SNCA* intron 1 in the frontal cortex shows a trend towards an increase in DNA methylation at all sites in idiopathic PD and four CpG sites in PD-*GBA1* compared with elderly non-PD controls. n=11 for PD-non*GBA1*, n=10 for PD-*GBA1*, n=7 for controls. Legend: PD-non*GBA1*=idiopathic PD. Error bars – SD. Corrected methylation percentage adjusted for age and gender.

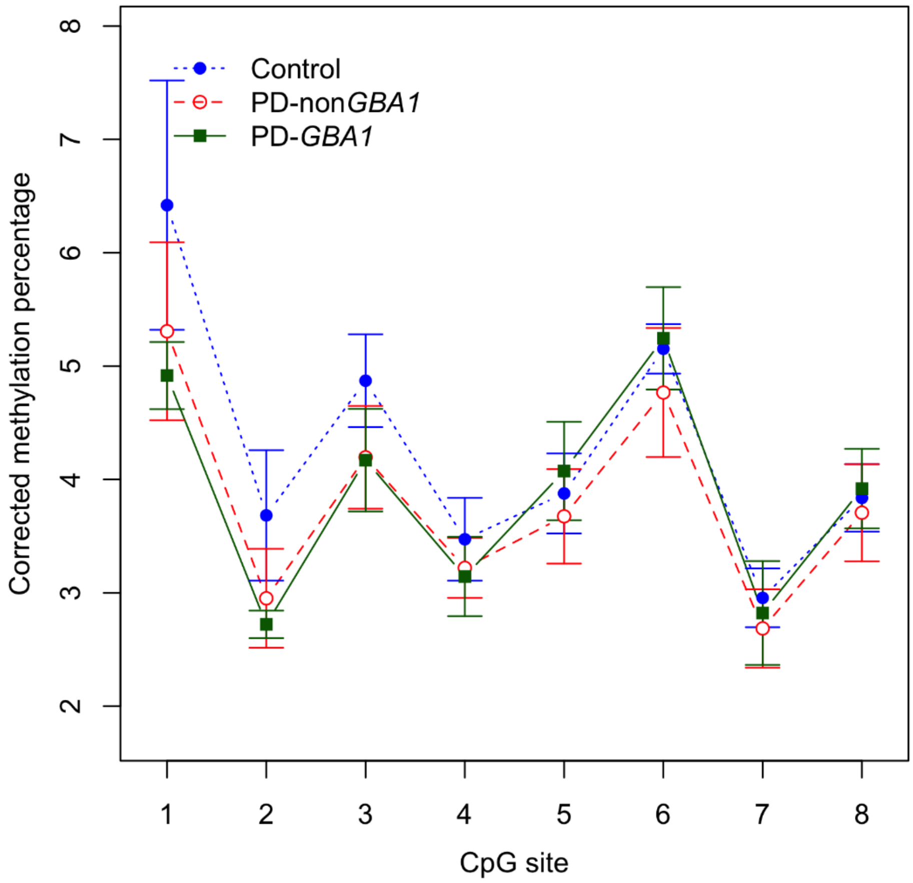


**Supplementary Figure S2: No significant DNA differences at eight consecutive CpG sites (CpG1-8) of *SNCA* intron 1 in the putamen in idiopathic PD.** The methylation profile within CpG1-8 of *SNCA* intron 1 in the putamen shows a trend towards a decrease in DNA methylation at all sites in idiopathic PD and five CpG sites in PD-*GBA1* compared with elderly non-PD controls. n=9 for PD-non*GBA1*, n=6 for PD-*GBA1*, n=6 for controls. Legend: PD-non*GBA1*=idiopathic PD. Error bars – SD. Corrected methylation percentage adjusted for age and gender.

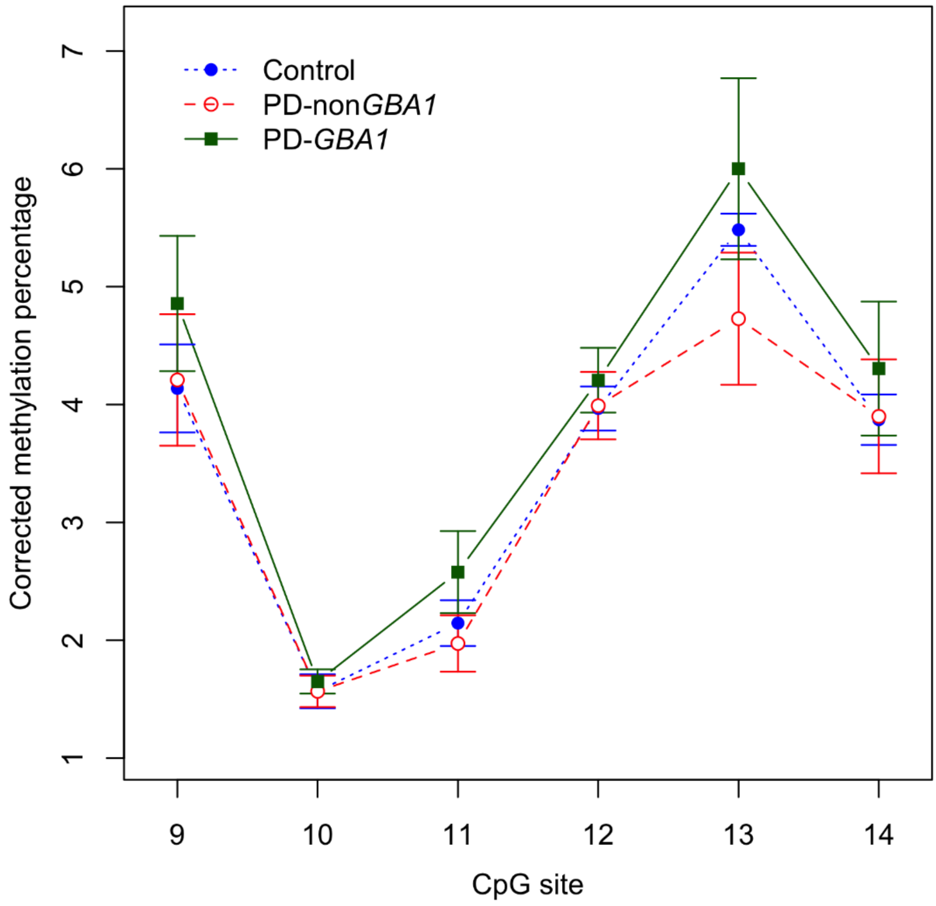


**Supplementary Figure S3: No significant DNA differences at a further six consecutive CpG sites (CpG9-14) of *SNCA* intron 1 in the putamen in PD-*GBA1*.** The methylation profile within CpG9-14 of *SNCA* intron 1 in the putamen shows a trend towards an increase in DNA methylation at all sites in PD-*GBA1* and four CpG sites in idiopathic PD compared with elderly non-PD controls. n=9 for PD-non*GBA1*, n=6 for PD-*GBA1*, n=6 for controls. Legend: PD-non*GBA1*=idiopathic PD. Error bars – SD. Corrected methylation percentage adjusted for age and gender.

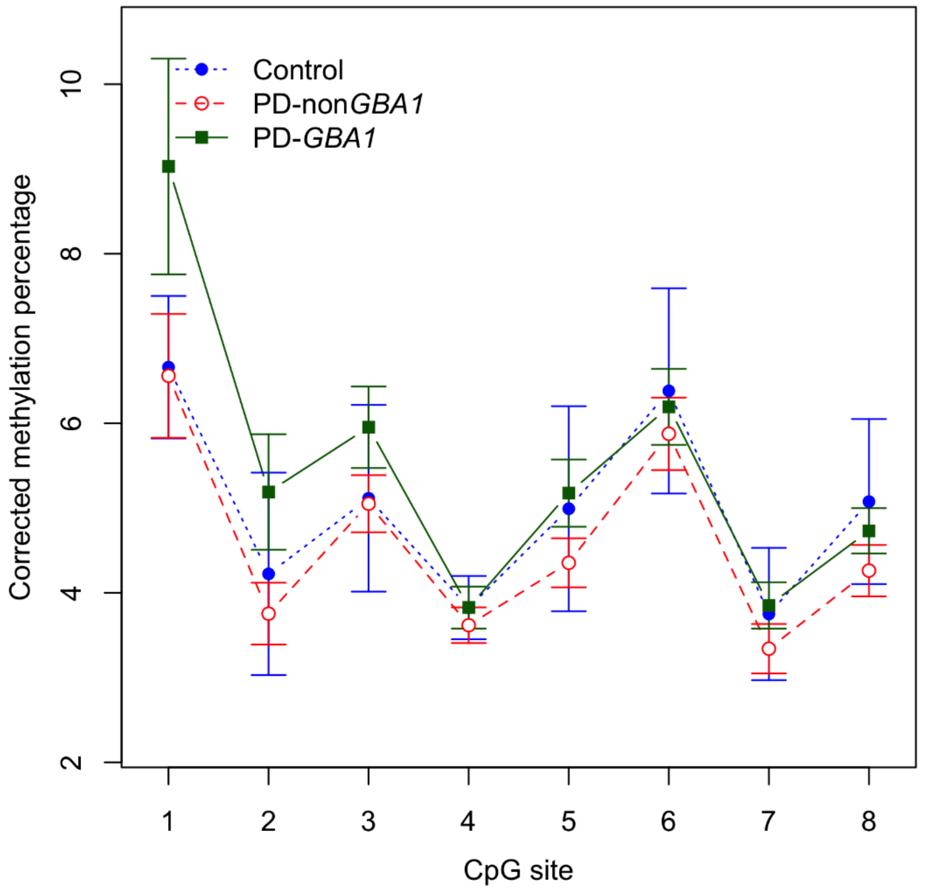


**Supplementary Figure S4: No significant DNA hypomethylation at eight consecutive CpG sites (CpG1-8) of *SNCA* intron 1 in substantia nigra in idiopathic PD.** The methylation profile within CpG1-8 of *SNCA* intron 1 in the substantia nigra shows a trend towards a decrease in DNA methylation at all sites in idiopathic PD and a trend towards an increase in DNA methylation six CpG sites in PD-*GBA1* compared with elderly non-PD controls. n=13 for PD-non*GBA1*, n=8 for PD-*GBA1*, n=3 for controls. Legend: PD-non*GBA1*=idiopathic PD. Error bars – SD. Corrected methylation percentage adjusted for age and gender.

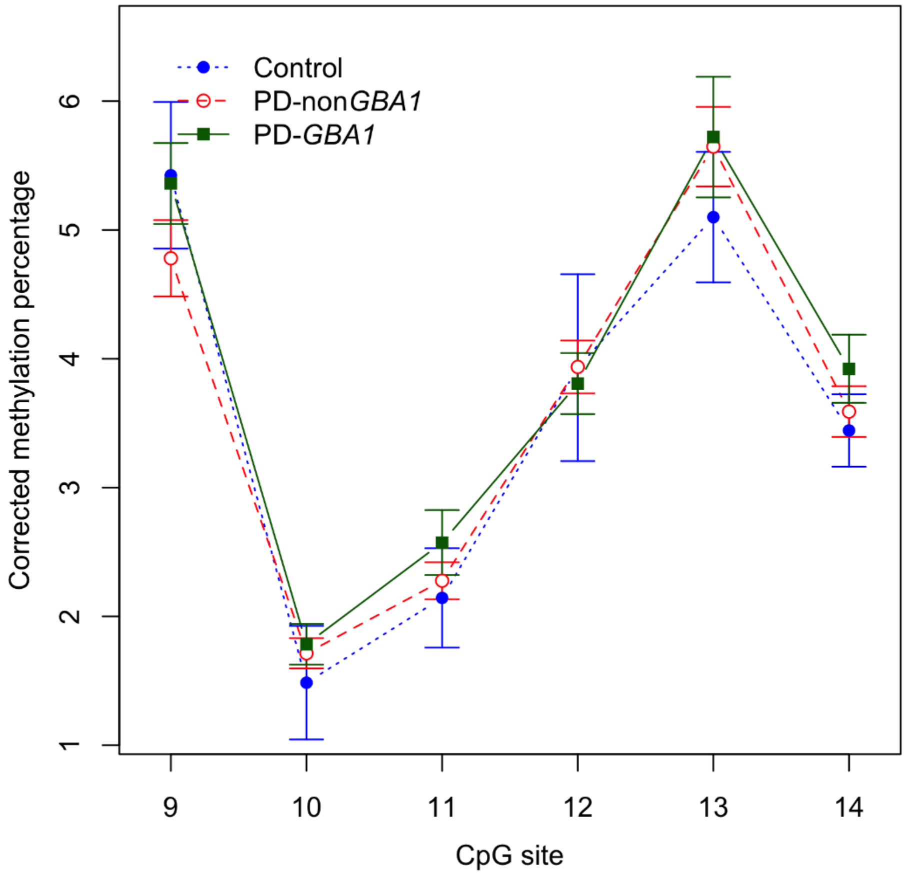


**Supplementary Figure S5: No significant DNA differences at a further six consecutive CpG sites (CpG9-14) of *SNCA* intron 1 in the substantia nigra in PD-*GBA1* and idiopathic PD.** The methylation profile within CpG9-14 of *SNCA* intron 1 in the substantia nigra shows a trend towards an increase in DNA methylation at five sites in PD-*GBA1* and four CpG sites in idiopathic PD compared with elderly non-PD controls. n=13 for PD-non*GBA1*, n=8 for PD-*GBA1*, n=3 for controls. Legend: PD-non*GBA1*=idiopathic PD. Error bars – SD. Corrected methylation percentage adjusted for age and gender.

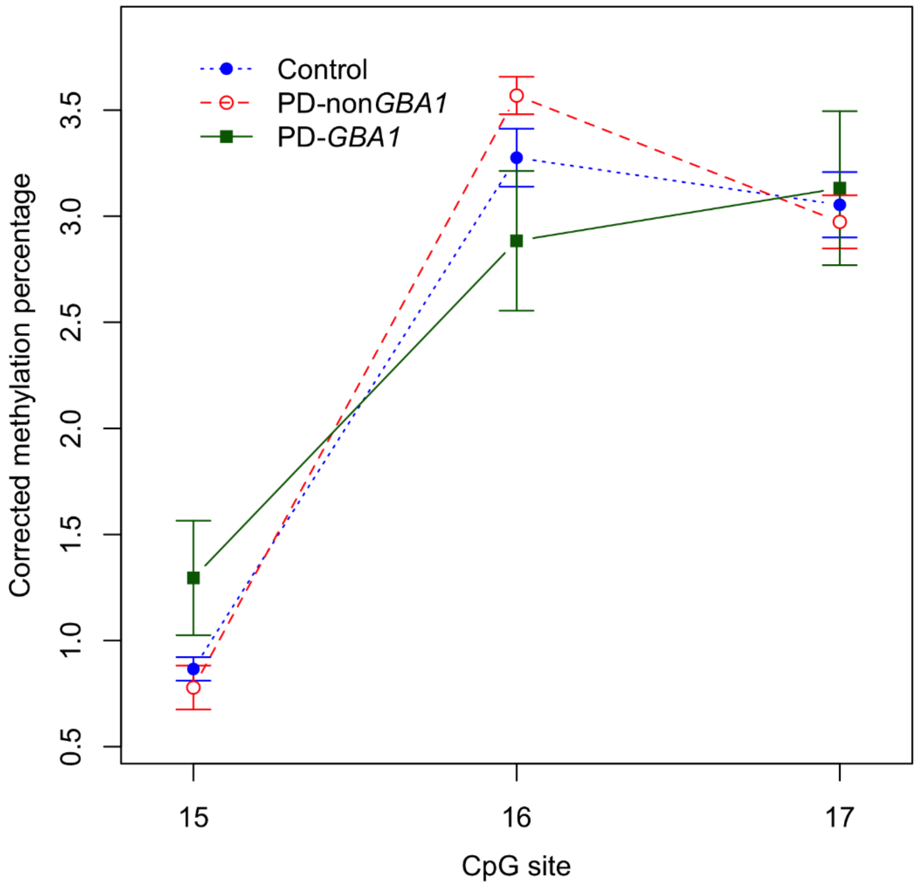


**Supplementary Figure S6: No significant DNA differences within the *SNCA* promoter in the frontal cortex in PD-*GBA1* and idiopathic PD.** n=11 for PD-non*GBA1*, n=10 for PD-*GBA1*, n=7 for controls. Legend: PD-non*GBA1*=idiopathic PD. Error bars – SD. Corrected methylation percentage adjusted for age and gender.

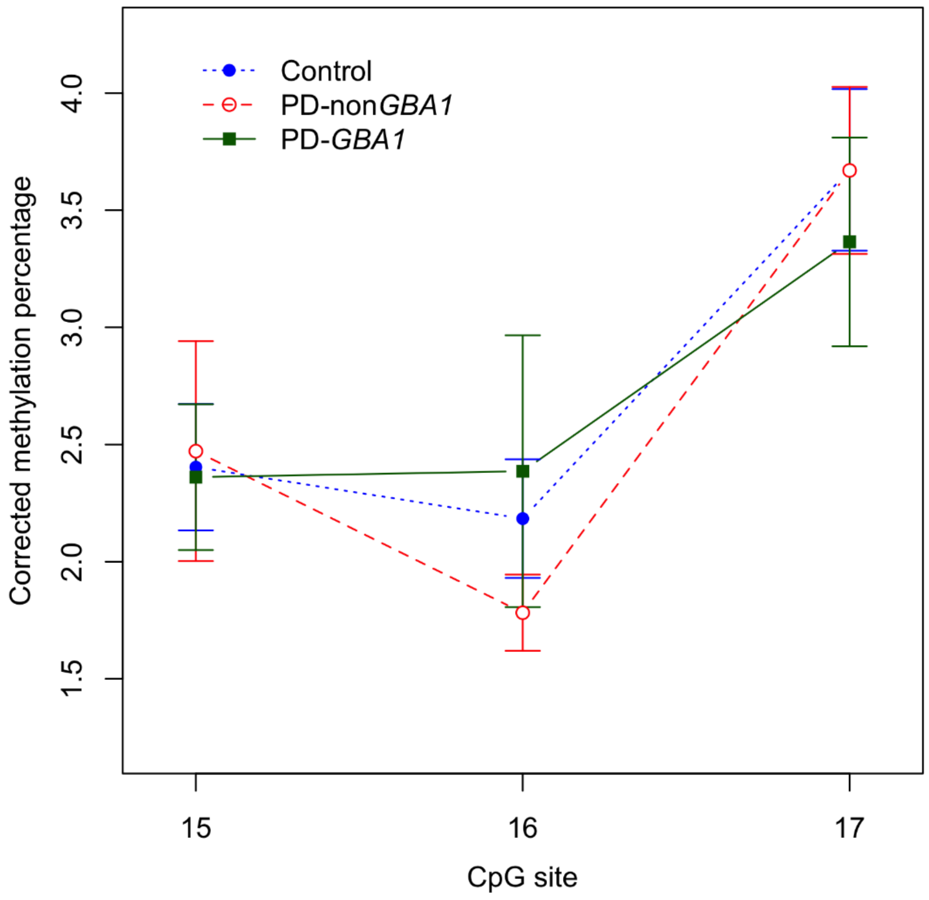


**Supplementary Figure S7: No significant DNA differences within the *SNCA* promoter in the putamen in PD-*GBA1* and idiopathic PD.** n=9 for PD-non*GBA1*, n=6 for PD-*GBA1*, n=6 for controls. Legend: PD-non*GBA1*=idiopathic PD. Error bars – SD. Corrected methylation percentage adjusted for age and gender.
